# Impact of climate change on the global circulation of chikungunya virus: current evidence, future projections, and adaptation strategies

**DOI:** 10.1186/s40249-026-01480-3

**Published:** 2026-07-24

**Authors:** Chao Wang, Xinyao Li, Lili Wang, Xiaohui Zhao, Yan Liu, Jianhua Xiao, Haoran Wang

**Affiliations:** 1 Department of Veterinary Surgery, Northeast Agricultural University, Harbin, Heilongjiang 150030 People’s Republic of China; 2 Heilongjiang Provincial Key Laboratory of Pathogenic Mechanism for Animal Disease and Comparative Medicine, Harbin, 150030 People’s Republic of China

**Keywords:** Chikungunya virus, Climate change, Adaptation strategy, Vector-borne pathogen, Arbovirus

## Abstract

**Background:**

Climate change is accelerating the circulation of the chikungunya virus (CHIKV), posing a significant threat to both endemic areas and immunologically naive temperate regions. This study aims to synthesize empirical climatic drivers, future transmission projections, and global adaptation strategies to evaluate current evidence and identify key research gaps.

**Methods:**

This scoping review completed protocol registration on the Open Science Framework prior to literature retrieval, with systematic searches conducted across PubMed, Web of Science, Scopus and EBSCOhost to collect all relevant English peer-reviewed papers published between Jan 1, 2000 and Nov 1, 2025. Multi-level screening filtered out unqualified literature, and unified datasets related to epidemiological parameters, predictive models and adaptation measures were extracted from 104 eligible studies.

**Results:**

The results indicate that: (1) Current evidence reveals post-2014 research favors Europe and South America, disproportionately focusing on temperature (*n* = 38) and precipitation (*n* = 32). CHIKV transmission exhibits a non-linear thermal optimum (23–30 °C) and a 1-to-4-week lag following extreme precipitation. (2) Future projections (*n* = 24) consistently indicate transboundary vector expansion into higher latitudes and altitudes. (3) Regarding adaptation strategies, we conceptualized a three-tiered intervention framework. However, a stark socioeconomic-climatic gap emerged: while 43% of the research centers on mid-tier early warning systems in high and upper-middle-income nations, evidence for the foundational infrastructural resilience in low-income regions is limited.

**Conclusion:**

This global climate-CHIKV synthesis reveals structurally imbalanced preparedness. To mitigate escalating transboundary risks, international health policies must pivot from solely reactive surveillance. Future priorities should include proactive, climate-resilient urban infrastructure and equitable cross-border coalitions in neglected low-resource settings.

**Graphical Abstract:**

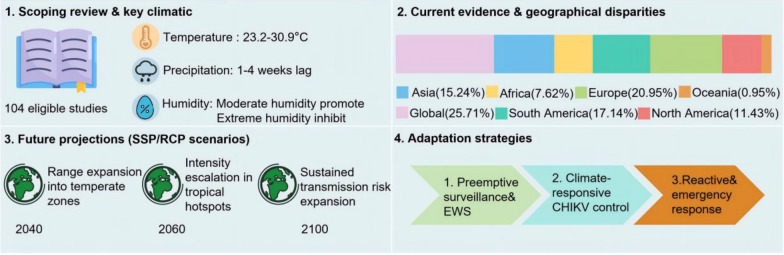

**Supplementary Information:**

The online version contains supplementary material available at 10.1186/s40249-026-01480-3.

## Background

Climate change imposes profound and escalating impacts on human health, fundamentally altering the geographical dissemination of vector-borne diseases [1]. Among these emerging threats, chikungunya virus (CHIKV)—an arbovirus of the genus *Alphavirus*—warrants urgent attention [2]. Primarily transmitted by *Aedes aegypti* and *Ae. albopictus* mosquitoes, CHIKV was historically confined to endemic pockets in tropical regions following its first documentation in Tanzania during the 1950s [3, 4] . However, driven by climate change and escalating global mobility, its transboundary expansion has significantly expedited [4]. For instance, the CHIKV epidemic in the Americas, which began in December 2013, continued to spread and affect new regions as case numbers increased and temperatures rose in the Northern Hemisphere [5]. Its epidemiological chain originated in Africa and has gradually expanded to Southeast Asia, South Asia, Indian Ocean islands, and the Americas [6–9]. This global epidemic outbreak occurred in July 2025; by the end of August, Foshan, Guangdong, China, reported more than 10,000 confirmed cases, setting a record for the largest outbreak in the country [10]. To date, autochthonous transmission of chikungunya fever has been reported in more than 100 countries and territories worldwide.

The global expansion of CHIKV is closely linked to the proliferation of its mosquito vectors, which are highly susceptible to environmental conditions [11]. Existing evidence strongly suggests that elevated temperatures accelerate viral replication, significantly reduce the extrinsic incubation period of the virus in mosquitoes, and improve transmission efficiency [12, 13]. Furthermore, extreme precipitation and El Niño–Southern Oscillation-induced climate events create optimal ecological niches for vector reproduction [14]. For example, prior to the 2025 Foshan outbreak, the region experienced abnormally conducive climatic conditions: days suitable for mosquito propagation cumulatively reached 38, with a peak period persisting for 23 days, thereby drastically elevating the local transmission risk [15]. Also, in temperate regions of Europe and North America, driven by climate change, the disease is expected to further spread northward and eastward, as well as to higher altitudes [16].

Beyond its rapid geographical expansion, the profound socioeconomic and disease burden inflicted by CHIKV necessitates urgent global intervention [17]. While generally self-limiting in mortality, CHIKV infection frequently transitions from acute high fever into chronic, incapacitating polyarthralgia that can persist for months or even years [18, 19]. This debilitating trajectory results in substantially high disability-adjusted life years, paralyzing local workforces and overwhelming healthcare systems, particularly in resource-limited settings [20]. Recognizing these severe health implications, the scientific community—catalyzed by milestone reports from the Intergovernmental Panel on Climate Change (IPCC) and the Lancet Commission on Climate Change and Health—broadly acknowledges that curbing this climate-induced burden requires robust adaptation strategies [21, 22]. However, current research remains highly fragmented. Critically, existing assessments frequently neglect the stark socioeconomic-climatic inequities inherent in these strategies, leaving highly vulnerable, low-income settings without an actionable evidence base for foundational infrastructural resilience.

To address these key knowledge and policy gaps and support evidence-based decision-making for global health authorities, this study synthesizes global evidence on climate-driven chikungunya virus transmission dynamics. Based on a scoping review of literature from 2000 to 2025, we aim to: (1) summarize existing evidence on thermal and precipitation thresholds linked to CHIKV transmission and identify geographic research disparities; (2) assess future projections of CHIKV and vector range expansion under climate change; (3) develop a structured, evidence-based adaptation framework to guide a shift from reactive surveillance toward proactive, climate-resilient global preparedness.

## Methods

### Study design and protocol registration

This scoping review was conducted and reported in strict adherence to the Preferred Reporting Items for Systematic reviews and Meta-Analyses (PRISMA) Extension for Scoping Reviews guidelines [23, 24, 25, 26]. A completed PRISMA 2020 Scoping Review checklist is provided as part of the Additional file 1 to ensure transparency and completeness of reporting. To ensure methodological transparency and reproducibility, the a priori protocol was prospectively registered with the Open Science Framework (https://osf.io/dq3h9/overview) on October 9, 2025.

### Search strategy

We systematically searched four major electronic databases: PubMed, Web of Science, Scopus, and EBSCOhost. Peer-reviewed literature published between January 1, 2000, and November 1, 2025 was included. The search strategy integrated two core sets of terminology: climate change parameters (adapted from the established bibliometric analysis by Sweileh, 2020) and CHIKV-specific identifiers, including: “climat* change” OR “global warming” OR “changing climate” OR “climate variability” OR “greenhouse gas” OR “rising temperature” OR “extreme weather” OR “greenhouse effect” [27]. CHIKV-specific search terms encompassed: “Chikungunya virus” OR “CHIKV host” OR “CHIKV vector”. While initial queries were conducted without language restrictions to maximize search sensitivity, final inclusion was restricted to English-language publications. The comprehensive search algorithms tailored for each database are detailed in Additional file 2.

### Eligibility criteria

Eligibility was defined using the Population, Concept, and Context framework.

#### Inclusion criteria

We included original observational, modelling, or experimental studies that: quantified the impacts of meteorological or climatic factors on CHIKV emergence, transmission, or geographic spread; or evaluated climate adaptation measures and policies targeting these risks. Specifically, observational studies included cohort, cross-sectional, time-series, and ecological designs; modelling studies included ecological niche models and mathematical predictive models; experimental studies included laboratory vector competence assays and field intervention trials.

#### Exclusion criteria

Studies were excluded if they: were non-original research (reviews, theses, conference abstracts) or preprints; focused only on routine mosquito control without explicit linkages to climate; or assessed climate effects on vectors without direct associations with CHIKV epidemiological outcomes.

### Study selection and data extraction

Citation management and deduplication of the initially identified records were performed using NoteExpress 3.8.0 (Beijing Aegean Software Co., Ltd., Beijing, China). Following deduplication, two independent researchers screened the titles and abstracts. Full texts of potentially relevant articles were then thoroughly evaluated against the eligibility criteria. Any discrepancies were resolved through consensus discussions involving a third senior reviewer. The detailed screening process, including specific reasons for inclusion and exclusion at each stage, is transparently documented in Additional file 2.

A pilot test of the data extraction framework was conducted on five randomly selected articles before formal extraction. Subsequently, data from the 104 ultimately eligible studies were systematically extracted into a comprehensive Microsoft Excel database (Additional file 3). Data fields encompassed: (1) bibliographic identifiers; (2) study characteristics (research design, geographic scope, target population); (3) key evidence regarding climate-driven impacts and documented adaptation measures.

### Evidence extraction and analysis

To holistically capture the climate-CHIKV associations, we structured our data synthesis around two distinct thematic domains: current empirical evidence (capturing thermal-precipitation thresholds and spatiotemporal lag effects) and future trajectories and resilience (extracting data from predictive models and adaptation strategies). A mixed-methods synthesis was applied to integrate quantitative and qualitative evidence. We extracted numerical data including climatic thresholds, spatiotemporal lags, and model parameters, alongside textual information on public health interventions, adaptive strategies, and climate-driven transmission mechanisms. These quantitative and qualitative findings were triangulated to form a balanced, comprehensive synthesis.

For descriptive spatial analysis, the geographic focus of the included articles was categorized into six distinct regions (North America, South America, Europe, Africa, Asia, and Oceania), and multi-regional investigations were classified as global. Concurrently, a thematic synthesis approach was employed to conceptualize the extracted interventions into a comprehensive, tiered adaptation framework. The thematic categories were deductively derived from the Global Arbovirus Initiative (GAI) framework to ensure alignment with authoritative global health standards. Two researchers independently performed data extraction and thematic coding; any disagreements were resolved via group discussion and cross-verification until consensus was achieved. All thematic synthesis was conducted manually with no qualitative analysis software involved. Adaptation strategies included in this study were classified into six major domains based on the March 31, 2022, launch report of the GAI by the World Health Organization (WHO), including monitor risk and anticipate, strengthen vector control, reduce epidemic risk, enhance innovation and new approaches, prevent and prepare for pandemics, and build a coalition of partners [28].

### Quality assessment

While optional for scoping reviews, we assessed the methodological quality of the included articles using the Joanna Briggs Institute Critical Appraisal Checklist to ensure evidence robustness [29]. Each study was evaluated against 8 items, with 1 point awarded for fulfillment of each criterion. Total scores ranged from 0 to 8, and studies were classified as low (0–2), moderate (3–5), or high (6–8) quality. The results are presented in Additional file 4.

### Gaps analysis

Through this scoping review, several critical gaps in knowledge and policy have been identified, underscoring the need for more comprehensive research to address the challenges posed by CHIKV under changing climatic conditions. One significant gap lies in the geographic disparities in existing studies, with a disproportionate focus on regions like South America and Asia, while areas such as Africa, Oceania, and multi-regional/global contexts are notably underrepresented. Another notable deficiency is the limited evidence on the success and implementation of climate adaptation strategies specifically targeting CHIKV risks, further emphasizing the importance of intervention trials and policy evaluations.

Moreover, very few studies have considered delayed or cumulative climate effects over extended time horizons, which are crucial for understanding long-term transmission trends. There is also a scarcity of data examining how CHIKV dynamics interact with co-endemic arboviral diseases such as dengue and Zika, interactions that are vital for comprehensively assessing epidemic risks. Furthermore, research on the feasibility and scalability of adaptation strategies, particularly for resource-constrained regions, remains largely unexplored. Addressing these gaps will require interdisciplinary, regionally inclusive, and methodologically innovative approaches to inform proactive and evidence-based climate-CHIKV adaptation strategies on a global scale.

## Results

### Characteristics of included studies

**Fig. 1:**
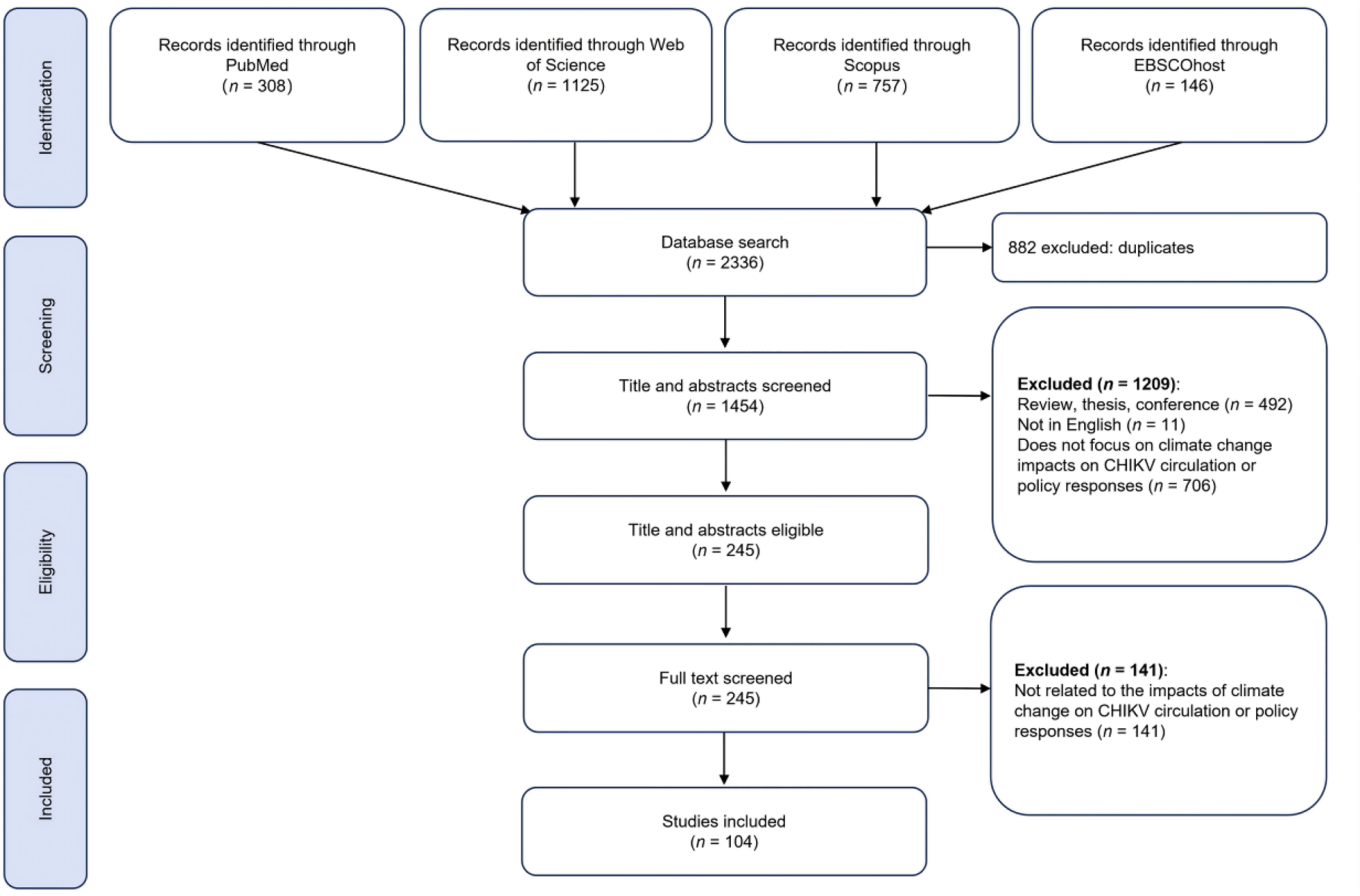
Flowchart diagram illustrating the study search and selection process for the scoping review of climate-associated CHIKV research. *CHIKV* chikungunya virus

Our literature search identified 2336 initial records. Following the removal of 882 duplicates using NoteExpress, we screened the titles and abstracts of the 1454 remaining records. This initial screening excluded 1209 irrelevant articles. We then evaluated the full texts of the 245 remaining articles against our eligibility criteria and excluded another 141 documents. Ultimately, our final synthesis included 104 eligible studies published between 2000 and 2025 (Fig. 1). Within this evidence base studies quantifying the direct impacts of climate change on CHIKV outcomes (*n* = 56) slightly outnumbered those exploring broader climatic associations (*n* = 48).

### Temporal trajectories

**Fig. 2:**
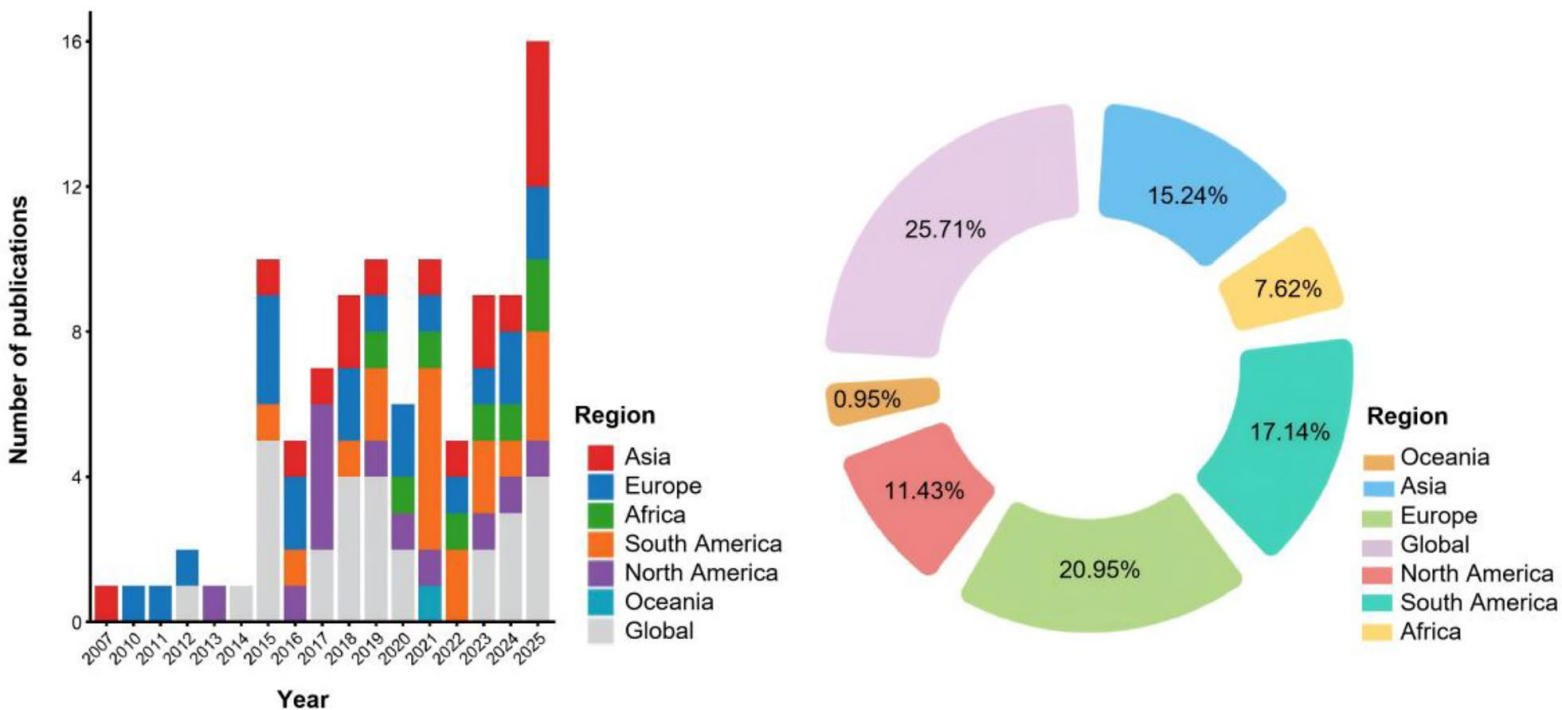
Annual publication number and geographical distribution of the included studies

A timeline analysis shows a clear change in research output (Fig. 2). Between 2000 and 2014, the number of studies was small, with only seven published articles. In contrast, 97 studies were published after 2014 resulting in a sharp upward trend. This increase closely follows the release of the IPCC Fifth Assessment Report in 2014 and the Lancet Commission on Health and Climate Change in 2015 [21, 22]. These reports acted as key drivers. They encouraged global scientific efforts to measure the health consequences of climate change and brought CHIKV into the focus of global health research. Moreover, the 2025 data show an increase in research focusing on Asia. This recent growth directly reflects new disease patterns. It is driven by recent outbreaks and the spread of the virus into subtropical and temperate Asian countries [30].

### Geographic distribution

Mapping the studies geographically shows an uneven distribution of research (Fig. 2). After excluding global scale modeling studies (*n* = 27) the empirical research is mostly located in Europe (*n* = 22) and South America (*n* = 18). This pattern matches recent outbreak events and the presence of better disease surveillance systems in these regions. However regions with a long history of the virus have received less attention. The number of studies is lower in Asia (*n* = 16) North America (*n* = 12) Africa (*n* = 8) and Oceania (*n* = 1).

The high number of studies in Europe and South America likely results from increased research funding after their recent local outbreaks. In contrast the lower research output from Africa and parts of Asia creates a clear knowledge gap. Africa is the historical epicenter of the virus, where climate conditions and healthcare resources should have been better documented and evidenced. However, the lack of local data from this region limits our capacity to develop accurate global predictive models and hinders the design of targeted public health strategies for populations that have endured the disease for the longest time [31].

### Socioeconomic gap

**Fig. 3:**
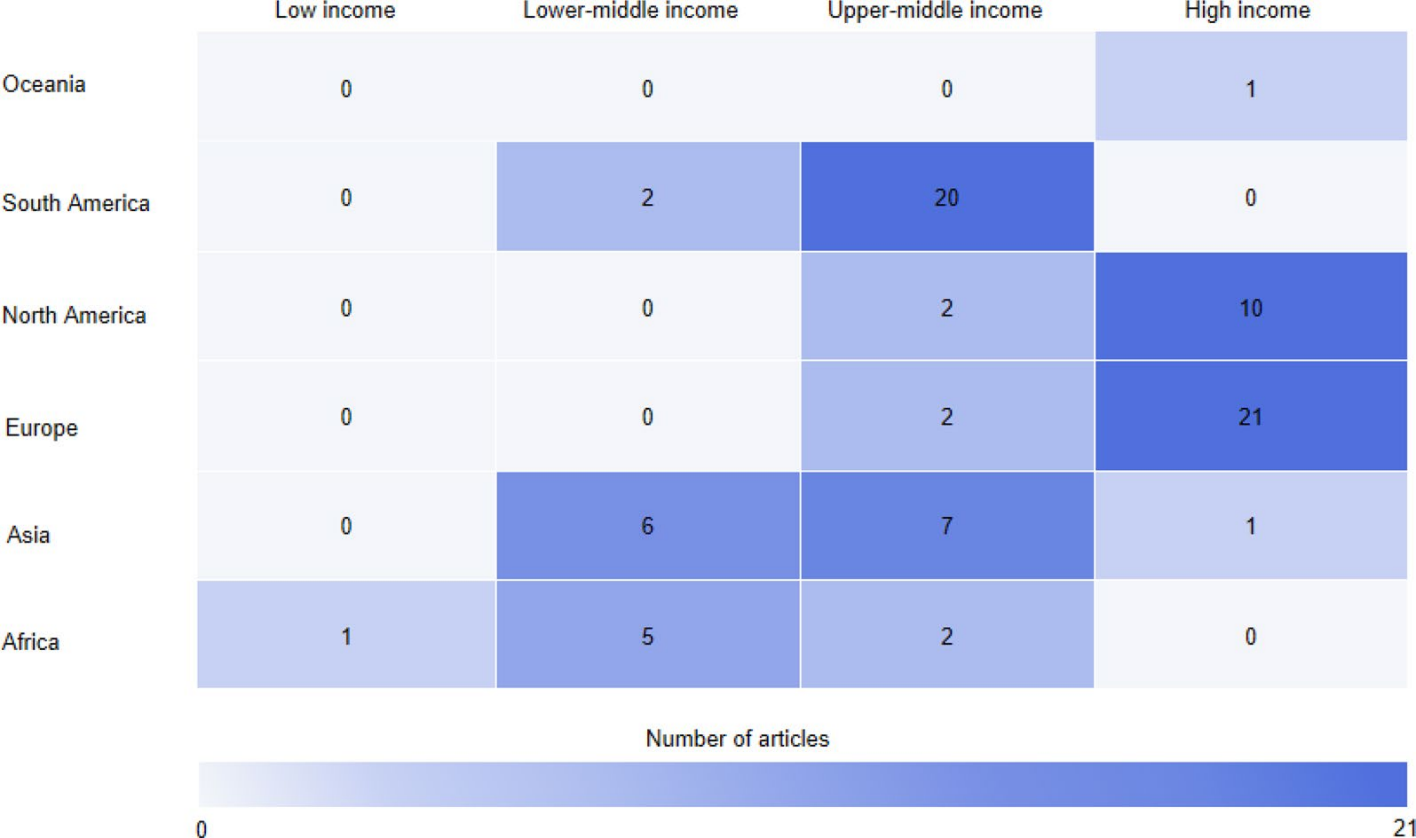
Distribution of included studies by World Bank income classification

Grouping the studies by World Bank income levels shows a clear economic difference in research output (Fig. 3). Most studies come from high income and upper-middle income countries. At the same time the number of publications from low income and lower-middle income countries was very limited. This economic gap creates a practical problem for global health [32]. Countries with lower incomes often face a higher burden of mosquito borne diseases but lack the local research needed to guide their health policies. Much of the current climate and health data comes from wealthier nations. However these data cannot be easily applied to lower income settings due to different living conditions and healthcare resources. Without local data these vulnerable regions struggle to develop effective climate adaptation plans. This situation shows a clear need to improve research funding and scientific capacity in lower income settings to better manage future viral outbreaks [17].

### Climate‑CHIKV associations

#### Temperature

**Fig. 4:**
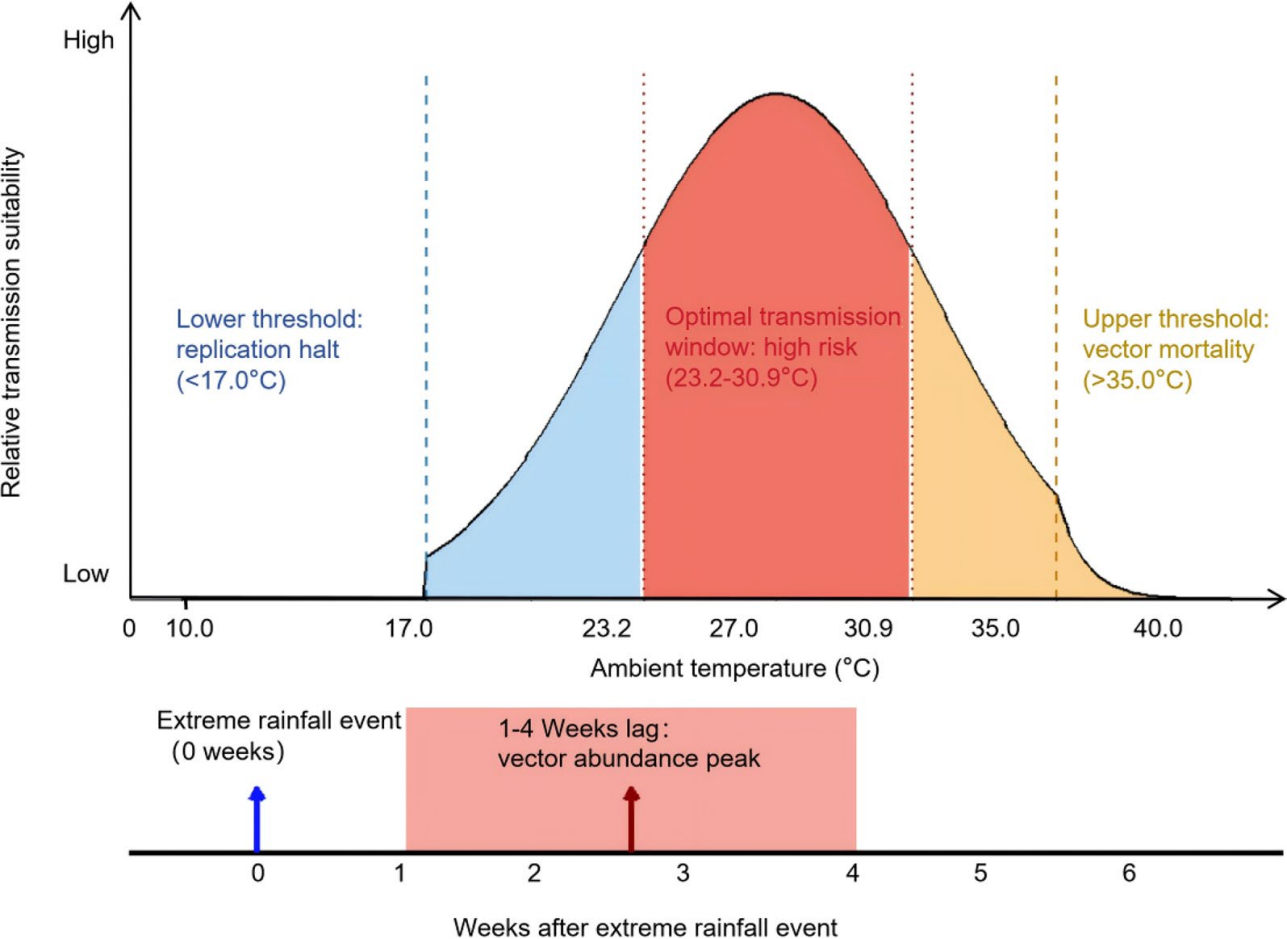
Mechanistic thresholds of temperature and precipitation for chikungunya virus transmission based on included literature in this review. The top panel shows the nonlinear relationship between ambient temperature and relative transmission suitability. The bottom panel illustrates the biological time lag between extreme rainfall events and peak vector abundance

Among the 48 articles investigating climate factors, 38 studies focus on ambient temperature, identifying it as the most fundamental driver of viral transmission. Within this group, 36 studies report a positive correlation, while two document a negative correlation. Temperature exerts a direct regulatory effect on both mosquito populations and viral kinetics. Our aggregated data reveals a consolidated optimal temperature window between 23 °C and 30 °C (Fig. 4, detailed data are provided in Additional file 5). This temperature range was derived by synthesizing evidence from these studies, with thresholds reported in each paper being tallied and assessed for consistency. Although no formal meta-analysis was conducted, we used a narrative summary approach to identify the range most frequently observed across diverse study designs and geographic settings. Within this specific thermal range, elevated temperatures increase mosquito survival rates, accelerate vector population growth, and shorten the extrinsic incubation period of the virus. This rapid biological development extends the seasonal window for local transmission [33–35]. Conversely, temperatures falling outside this optimal range severely restrict viral spread. When the ambient temperature drops below 17 °C, the vector competence of mosquitoes will decrease significantly [36]. The two studies reporting a negative correlation highlight the impact of extreme high temperatures exceeding 35 °C. This suppression occurs because extreme heat reduces mosquito survival and limits the catalytic activity of viral ribonucleic acid polymerase, an enzyme disruption that actively suppresses viral replication within the mosquito host [37, 38].

#### Precipitation

Of the 48 climate-related articles, 32 investigate the associations between rainfall and virus dynamics. The results reveal a complex dual effect on transmission as 15 studies report a positive correlation, 15 document a negative correlation, and two describe varying positive and negative correlations across different regions. Precipitation acts as the primary determinant of mosquito breeding habitats. On the one hand, moderate rainfall creates essential waterlogged environments and leads to an increase in vector mosquito populations [39, 40]. For instance, field investigations in tropical semi-arid regions demonstrate that positive anomalies in surface soil moisture directly boost mosquito vector populations and enhance local transmission potential [40]. On the other hand, extreme weather events drive negative correlations. Heavy storms can flush out established mosquito habitats. This phenomenon is particularly evident in high-altitude montane ecosystems where total rainfall exerts a significant inhibitory effect on transmission [41]. Similarly, insufficient rainfall also suppresses disease spread by desiccating standing water sources. This process leads to a reduction in available oviposition sites as documented in Mediterranean climatic zones [42, 43]. Despite these ecological variations, epidemiological data consistently confirm a clear lag period of typically 1–4 weeks between rainfall events and spikes in human infection (Fig. 4) [39, 44–47].This temporal range was derived through a narrative summary of findings across the included studies. We tallied and reviewed the lag intervals reported in each study, accounting for differences in methodology and climatic settings, and identified 1–4 weeks as the most commonly reported range. No formal meta-analysis was performed; instead, the interval represents the predominant trend observed across diverse contexts.

#### Humidity

Humidity is explored in 11 out of the 48 climate-related articles. Among these eight studies report a positive correlation and three document a negative correlation. Humidity serves as a critical synergistic factor that modifies the local microclimate for vector survival and reproduction [48]. The studies reporting a positive correlation highlight the role of moderate to high humidity in maintaining mosquito populations. Evidence from Iran shows that ambient humidity directly extends the lifespan of breeding sites and elevates larval survival rates [49]. Complementary research in Spain confirms that higher humidity levels lead to a marked increase in mosquito egg production [50]. However specific geographic areas present negative correlations. These suppressive effects usually occur when humidity exceeds the natural adaptive range of the local vectors [48]. For instance, research in southern Brazil indicates that persistently high relative humidity (above 70%) falls outside the optimal survival range of dominant local mosquito species [43]. It is worth noting that all studies reporting negative correlations are concentrated in mid-to-high latitudes or regions with large humidity fluctuations, which suggests that humidity’s inhibitory effect is related to exposure to non-optimal humidity ranges, rather than being a direct negative impact of humidity itself [43, 48]. Given the small number of studies documenting negative associations, this interpretation should be regarded as preliminary and context-specific.

### Climate change impacts on CHIKV

#### Current evidence

Of the 56 climate change-focused studies, six provide direct empirical evidence that changing weather patterns are already altering the epidemiology of the virus. These studies confirm a clear shift in both transmission intensity and geographical distribution. Research in southern China links recent local outbreaks to prolonged humid subtropical conditions. Specifically, environments maintaining average temperatures between 26 °C and 29 °C greatly increased mosquito activity and human infection rates [15]. Evidence from Brazil shows similar environmental amplifiers in tropical savanna regions. Researchers found that simultaneous heatwaves and irregular rainfall cycles boosted transmission intensity by 30% compared to periods with stable weather [51]. Beyond transmission intensity, climate change also drives the expansion of vector habitats. In Ecuador, a time-series analysis between 1988 and 2024 identified a northward expansion of mosquito habitats by 45 km per decade. Rising winter temperatures reduced the cold-related mortality of mosquito eggs, allowing them to survive the winter. This habitat shift resulted in a 2.5-fold increase in locally acquired cases within newly colonized temperate highland regions [52].

#### Future projections

The remaining 50 studies focus on predicting future transmission risks. Ecological niche models serve as the primary tools for these predictions (Additional file 3). At the global scale, modeling studies predict that the risk of viral transmission will expand significantly by 2040, 2060, 2080, and 2100. This expansion occurs across all Shared Socioeconomic Pathways (SSP), including SSP1-2.6, SSP2-4.5, SSP3-7.0, and SSP5-8.5. These models forecast increased transmission in existing tropical hotspots [53, 54].

Regional projections reveal a clear shift toward higher latitudes and altitudes [55]. In Europe, studies predict new transmission risks in countries like Germany and Italy. Rising temperatures in these areas will likely extend the active periods for vectors and create suitable breeding conditions beyond current seasonal limits [56, 57]. In Oceania, predictive models show that the epidemic potential in Australia will rise by 2029, with eastern and northern coastal regions facing a higher risk of local outbreaks [58]. North American models echo this trend, projecting that Canada will experience suitable climatic conditions for local transmission by 2040 and 2070 under both Representative Concentration Pathway (RCP) 4.5 and RCP 8.5 scenarios [55].

### Adaptation strategies

**Fig. 5:**
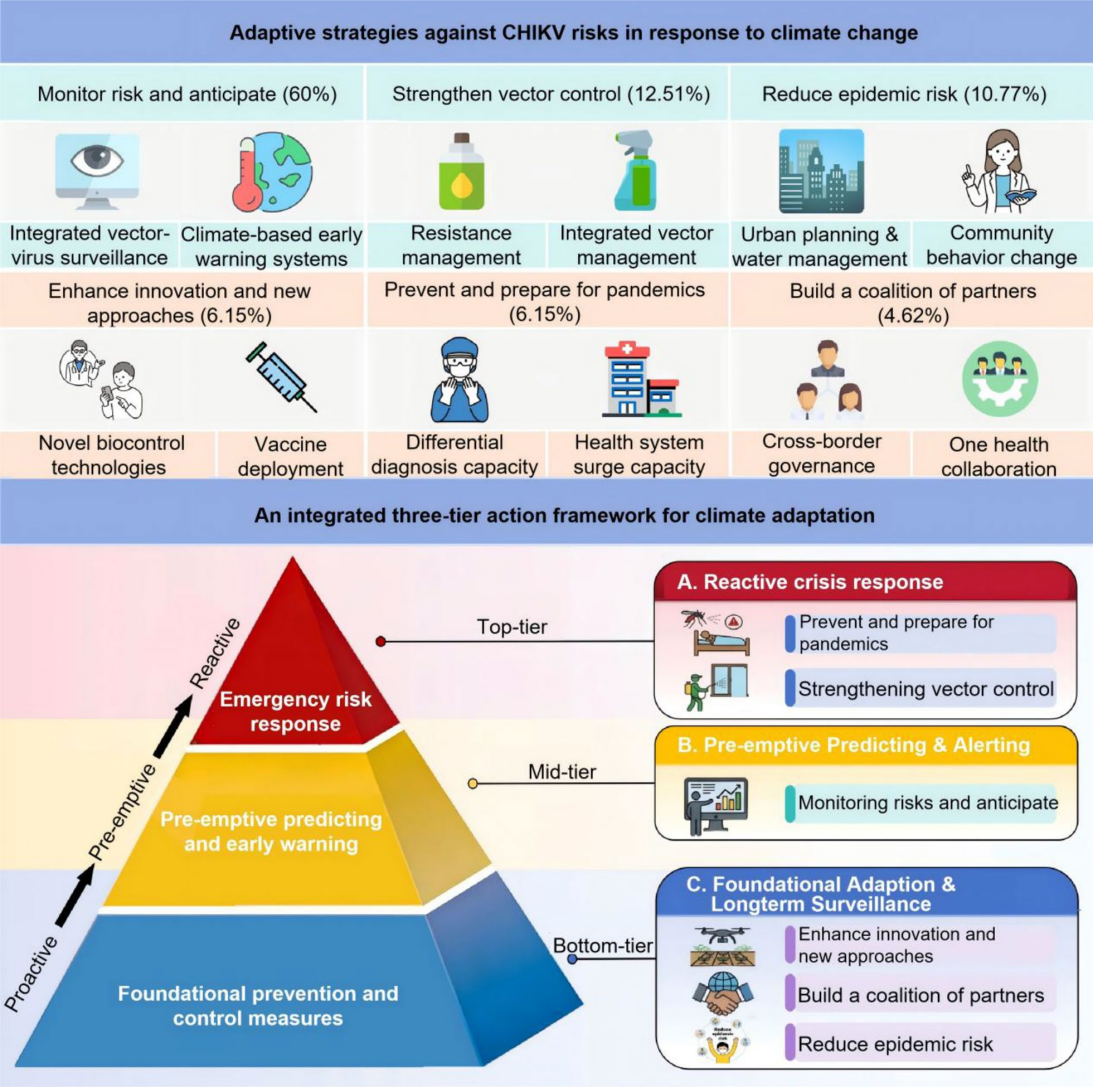
An integrated three-tier action framework for climate adaptation, transitioning from proactive foundational prevention to reactive emergency response. *CHIKV* chikungunya virus

Among the 104 included studies, 65 explicitly addressed adaptation strategies targeting CHIKV. Based on GAI, we classified these interventions into six main domains and further divided them into 12 specific sub-sectors (Additional file 6). To develop a clear strategic roadmap, we synthesized the core approaches from domains including six adaptive measures and constructed a three-tier action framework (Fig. 5). The three-tier framework proposed in this paper was developed through an inductive thematic analysis of the 104 included studies. Interventions were classified into bottom-, mid-, and top-tier strategies based on three key dimensions: temporal sequence (long-term prevention, mid-term monitoring, short-term emergency response), functional roles (prevention, surveillance, rapid response), and levels of systemic preparedness (foundational, intermediate, advanced). This categorization emerged directly from the evidence extracted from the reviewed studies, reflecting a logical continuum and the practical sequence of interventions in real-world contexts, rather than relying on any predefined criteria or adaptation from existing frameworks.

To enhance its policy relevance, the six domains identified by the GAI were subsequently mapped onto this evidence-based framework as follows: bottom-tier strategies correspond to long-term actions aimed at reducing epidemic risk, building a coalition of partners, and enhancing innovation and new approaches; mid-tier strategies address monitoring risk and anticipation; and top-tier interventions focus on preventing and preparing for pandemics and strengthening vector control. This alignment with WHO domains was integrated after the framework was established to improve its contextual applicability and practical interpretability. Importantly, it should be emphasized that this three-tier framework was not directly adapted from WHO frameworks or predefined at the outset. Rather, it was inductively derived based on thematic patterns and key insights identified within the included studies, with the alignment to WHO domains serving as a subsequent step to enhance its practical utility in global health policy contexts.

### Bottom-tier: foundational prevention and control

Studies in this foundational category (22% of the evidence base) focus on proactive systemic resilience. This tier encompasses reducing epidemic risk (seven studies), enhancing innovation (four studies), and building partner coalitions (three studies). To mitigate epidemic risk, countries rely on urban planning and water management to eliminate breeding sites. In Cambodia, studies indicate that urban heat amplification expands mosquito habitats into previously unsuitable areas, necessitating integrated heat mitigation and water planning [37]. In China, where erratic rainfall reduces maximum mosquito abundance, upgrading urban water infrastructure provides a sustainable solution to stabilize breeding site conditions [59]. Another critical component is community behavior change. While Italian health authorities implement targeted campaigns to eliminate standing water on private properties, educational programs in the United States focus on elucidating the environmental drivers of vector dynamics to improve community compliance [38, 60]. Furthermore, innovation strategies emphasize prioritizing vaccine development in regions projected to face expanded transmission [61]. Finally, building coalitions requires cross-sector collaboration among public health agencies, environmental departments, and local communities to avoid fragmented responses to the cross-regional spread of the virus [62].

### Mid-tier: predicting and early warning

Monitoring risk and anticipating outbreaks emerged as the predominant strategy, representing 60% of the documented approaches (39 out of 65 studies). This mid-tier relies on climate-based early warning systems and integrated surveillance. Early warning systems utilize disease modeling to identify spatiotemporal transmission risks. For example, researchers in Thailand integrated long-term meteorological data with incidence records to develop non-linear association models, identifying critical climatic thresholds for targeted alerts. Similarly, research in Iran applied geographic information system mapping to monitor occurrence trends by combining climate variables and local environmental characteristics [63]. Integrated surveillance involves the synchronous monitoring of vector populations and human cases. Studies in Germany and Italy recommend implementing entomological surveillance for established mosquito populations, paired closely with human case notification systems. This dual approach aims to detect early viral introductions and prevent autochthonous transmission [56]. Globally, surveillance efforts are prioritized in regions with high case uncertainty and emerging climatic suitability, such as the Middle East and Central Africa, to address critical monitoring gaps [64].

### Top-tier: emergency risk response

The top tier focuses on reactive emergency response, including strengthening vector control (eight studies) and preventing pandemics (four studies). To strengthen vector control, regions implement integrated management combining chemical and physical tools to minimize over-reliance on a single intervention. This approach is particularly critical in regions facing increased precipitation, which expands potential breeding sites [39, 65]. Studies in Brazil and Colombia demonstrate that aligning spraying schedules with climate-driven vector activity peaks maximizes efficacy, while targeted application mitigates environmental impacts [51, 66]. Resistance management is equally vital. Research in North America highlights that climate change can shorten vector generation cycles, accelerating resistance evolution in areas with frequent insecticide application [48]. Furthermore, studies in Mexico emphasize the necessity of monitoring local temperature fluctuations, which can temporarily alter mosquito insecticide susceptibility. Authorities must distinguish these temporary climate-induced variations from true genetic resistance to prevent the misadjustment of control measures [67]. Finally, pandemic preparedness requires rapid medical responses. Studies in Italy emphasize raising healthcare workers' awareness of clinical manifestations during summer transmission peaks to reduce diagnostic delays and contain immediate outbreaks [57].

## Discussion

### Summary of principal findings

This scoping review provides a comprehensive synthesis of the intricate relationships between climatic variability, CHIKV transmission dynamics, and global adaptation strategies. By systematically analyzing 104 studies, we identified a consistent one-to-four-week precipitation lag period and a reported thermal optimum for viral transmission that broadly ranges from 23 °C to 31 °C while being heavily modulated by regional ecology. Furthermore, our synthesis of predictive models indicates a significant future spatial expansion of CHIKV risk, with climate change potentially driving autochthonous transmission into higher latitudes and altitudes. Crucially, transitioning beyond epidemiological observations, we synthesized current vector control measures into a novel three-tier action framework. This framework highlights a critical paradigm shift required in global public health: moving from reactive emergency responses to proactive, climate-resilient foundational planning.

### Complex climate drivers

While the epidemiological dynamics of CHIKV are largely influenced by climate change, our review reveals a pronounced methodological bias in the current literature. Ambient temperature and precipitation overwhelmingly dominate the research focus. As evidenced by our structured summary of findings, the vast majority of empirical studies and predictive models anchor their risk assessments almost exclusively on thermal thresholds and rainfall anomalies. However, this binary focus risks oversimplifying the complex ecological web of vector-borne diseases. Other critical environmental variables remain significantly underexplored. For instance, while relative humidity directly dictates mosquito desiccation rates and survival longevity, it was investigated in only a minor fraction of the literature [68]. Furthermore, variables such as wind speed and direction, which facilitate the passive long-distance dispersal of vector populations, and microclimatic soil moisture, which defines the viability of breeding sites, are largely absent from current ecological models [69]. Future research must transition toward comprehensive, multi-dimensional climate modeling to refine the accuracy of spatiotemporal predictions [64].

### Geographic shifts and global health equity

The projected spatiotemporal expansion of CHIKV under future climate change scenarios underscores an impending global health crisis. Our findings consistently demonstrate that rising global temperatures and shifting precipitation patterns are expanding the ecological niche of *Aedes* vectors toward higher latitudes and altitudes. Predictive models under high-emission scenarios (SSP3-7.0, SSP5-8.5) forecast the emergence of autochthonous CHIKV transmission in previously non-endemic, temperate regions across North America, Europe, and Oceania by the mid-twenty-first century. However, these geographic projections must be interpreted with critical methodological caution [54]. As our narrative summary reveals, current ecological niche models predominantly over-fit to macro-climatic variables—specifically ambient temperature and gross precipitation—while systematically neglecting granular microclimatic drivers. The failure to incorporate critical regulating factors, such as relative humidity's impact on egg desiccation and microclimatic soil moisture dynamics, creates significant uncertainty. Consequently, current models may misrepresent the true localized colonization risk, potentially overestimating establishment in arid higher latitudes while underestimating it in micro-environmentally suitable refugia. Despite these methodological limitations, this anticipated geographic shift fundamentally challenges the traditional perception of CHIKV as a strictly tropical disease. Consequently, while tropical regions will continue to bear the heaviest burden due to intensified transmission cycles, healthcare systems in high-income temperate nations face an unprecedented vulnerability. The lack of population immunity and historical vector control preparedness in these newly colonized zones necessitates the urgent establishment of local entomological surveillance [70].

### Paradigm shift in adaptation strategies

In response to these escalating threats, the three-tier action framework developed in this review serves as a strategic roadmap for climate adaptation. Our analysis reveals that current global efforts are disproportionately concentrated in the mid-tier, with 60% of studies focusing on risk monitoring and early warning systems. While predictive surveillance is indispensable, it remains fundamentally a secondary prevention measure. To achieve equitable long-term systemic resilience, global health policies must urgently reallocate resources toward bottom-tier foundational prevention, recognizing that one-size-fits-all strategies are fundamentally flawed [71]. While climate-adapted urban planning and large-scale water infrastructure upgrades are viable for middle- to high-income nations, adaptation in neglected low-resource settings must be driven by implementation science. International financing must aggressively pilot and scale decentralized environmental management—such as community-led water, sanitation and hygiene integration and low-tech source reduction—rather than merely exporting unaffordable surveillance paradigms.

### Limitations and future directions

Several limitations of this scoping review must be acknowledged. First, the geographical distribution of the included literature exhibits a distinct bias, with a severe lack of empirical data from low-income nations in Sub-Saharan Africa, which often face high endemic burdens but lack research funding. Second, the considerable heterogeneity in climate modeling methodologies across studies precludes a formal meta-analysis of transmission risk parameters. Third, this review was restricted to English-language publications only. While this constraint was adopted for practical feasibility, it may introduce language bias and potentially limit the representativeness of findings from non-English-speaking endemic regions. In addition, the search strategy relied on keyword-based syntax without standardized subject headings in databases such as EBSCOhost, which may represent a further limitation in comprehensiveness. Future research should prioritize empirical field studies in these underrepresented regions and adopt standardized ecological niche modeling protocols. Ultimately, addressing the climate-driven expansion of CHIKV requires an integrated, cross-sectoral approach that embeds proactive environmental management into the core of global public health infrastructure.

## Conclusions

This review explores the impact of global climate change on CHIKV. Our comprehensive synthesis reveals that rising temperatures and extreme precipitation patterns not only intensify transmission within tropical endemic zones but also drive the transboundary expansion of the transmission vectors or CHIKV into higher-latitude and higher-altitude regions. To mitigate this escalating global health threat and address the stark socioeconomic-climatic inequities, we propose a novel three-tier action framework that integrates proactive foundational prevention and control, pre-emptive predicting and early warning, and reactive emergency risk response. Guided by this framework, we advocate for an urgent paradigm shift—transitioning global health priorities away from solely relying on reactive measures toward building long-term systemic resilience at the foundational level, especially for highly vulnerable, low-income regions. Achieving this intrinsically requires a robust One Health approach, fostering equitable cross-border and cross-sectoral collaboration to simultaneously address human vulnerability, vector ecology, and environmental sustainability [72]. Ultimately, embedding climate-adapted strategies into global public health infrastructure is imperative to prevent climate-driven CHIKV from becoming the next major pandemic.

## Supplementary Information

Supplementary Material 1

Supplementary Material 2

Supplementary Material 3

Supplementary Material 4

Supplementary Material 5

Supplementary Material 6

## Data Availability

The sources of all data extracted for this review can be found in Supplementary Materials.

## Abbreviations

*CHIKV*: Chikungunya virus
*PRISMA*: Preferred Reporting Items for Systematic reviews and Meta-Analyses
*GAI*: Global Arbovirus Initiative
*WHO*: World Health Organization
*IPCC*: Intergovernmental Panel on Climate Change
*SSP*: Shared Socioeconomic Pathways
*RCP*: Representative Concentration Pathway
