# Supplementary material for: Impact of climate change on the global circulation of chikungunya virus: current evidence, future projections, and adaptation strategies
Source: Infect Dis Poverty. 2026 Jul 24;15:83. doi: 10.1186/s40249-026-01480-3 (PMC13397683; doi:10.1186/s40249-026-01480-3)
Supplement: Supplementary file 1 — Supplementary Material 1 [file 40249_2026_1480_MOESM1_ESM.docx]

**Additional file 1. Search strategy by database**

**1. Key concepts**

| Climate change | Chikungunya virus |
| --- | --- |

**2. Associated keywords:**

| **Climate change** | **Chikungunya virus** |
| --- | --- |
| Climate | Chikungunya virus |
| Changing climate | Chikungunya fever |
| Global warming | CHIKV |
| Climate variability | Chikungunya disease |
| Climate fluctuation | Chikungunya virus infection |
| Rising temperature | CHIKV infection |
| Temperature | Arthralgia, Chikungunya fever |
| Precipitation | Chikungunya fever Arthralgia |
| Extreme weather | Chikungunya fever arthrodynia |
| Extreme event | Chikungunya fever arthrodynia |
| Extreme temperature | CHIKV transmiss* |
| Heat wave | CHIKV vector |
| Drought | CHIKV host |
| Flood | CHIKV circulation |
| Sea level rise |  |
| CO_2_ concentration |  |
| Greenhouse effect |  |
| Greenhouse gas |  |
| Anthropogenic forcing |  |
| Natural forcing |  |

And use these words (Transmiss* or Spread* or Circulat* or Distribut* or Disseminat* or Propagat* or Contagion* or Communicat* or Diffus* or Dispers* or Expans* or Outbreak* or Epidemic* or Pandemic* or Prevalen* or Inciden* or Occurren* or Emergen* or Clon* or Persisten* or Infect*) to limit the results.

**3. Databases to search:**

Scientific literature: PubMed, Scopus, Institute for Scientific Information Web of science, EBSCOhost

**Pubmed**

(Climat* [Title/Abstract] or Climate [Title/Abstract] or Changing climate [Title/Abstract] or Global warming [Title/Abstract] or Climate variability [Title/Abstract] or Climate fluctuation [Title/Abstract] or Rising temperature [Title/Abstract] or Temperature [Title/Abstract] or Precipitation [Title/Abstract] or Extreme weather [Title/Abstract] or Extreme event [Title/Abstract] or Extreme temperature [Title/Abstract] or Heat wave [Title/Abstract] or Drought [Title/Abstract] or Flood [Title/Abstract] or Sea level rise [Title/Abstract] or CO2 concentration [Title/Abstract] or Greenhouse effect [Title/Abstract] or Greenhouse gas [Title/Abstract] or Anthropogenic forcing [Title/Abstract] or Natural forcing [Title/Abstract])

and (Chikungunya Virus [Title/Abstract] or Chikungunya Fever [Title/Abstract] or CHIKV [Title/Abstract] or Chikungunya disease [Title/Abstract] or Chikungunya virus infection [Title/Abstract] or CHIKV infection [Title/Abstract] or Arthralgia, Chikungunya fever [Title/Abstract] or Chikungunya fever arthrodynia [Title/Abstract] or CHIKV transmiss* [Title/Abstract] or CHIKV vector [Title/Abstract] or CHIKV host [Title/Abstract] or CHIKV circulation [Title/Abstract])

and (Transmiss* [Title/Abstract] or Spread* [Title/Abstract] or Circulat* [Title/Abstract] or Distribut* [Title/Abstract] or Disseminat* [Title/Abstract] or Propagat* [Title/Abstract] or Contagion* [Title/Abstract] or Communicat* [Title/Abstract] or Diffus* [Title/Abstract] or Dispers* [Title/Abstract] or Expans* [Title/Abstract] or Outbreak* [Title/Abstract] or Epidemic* [Title/Abstract] or Pandemic* [Title/Abstract] or Prevalen* [Title/Abstract] or Inciden* [Title/Abstract] or Occurren* [Title/Abstract] or Emergen* [Title/Abstract] or Clon* [Title/Abstract] or Persisten* [Title/Abstract] or Infect* [Title/Abstract])

**Scopus**

(TITLE-ABS-KEY ( ("Climat*" or "Climate " or "Changing climate" or "Global warming" or "Climate variability" or "Climate fluctuation" or "Rising temperature" or "Temperature " or "Precipitation " or "Extreme weather" or "Extreme event" or "Extreme temperature" or "Heat wave" or "Drought" or "Flood" or "Sea level rise" or "CO2 concentration" or "Greenhouse effect" or "Greenhouse gas" or "Anthropogenic forcing" or "Natural forcing") ) ) and (TITLE-ABS-KEY ( ( "Chikungunya Virus" or "Chikungunya Fever" or "CHIKV" or "Chikungunya disease" or "Chikungunya virus infection" or "CHIKV infection" or "Arthralgia, Chikungunya fever" or "Chikungunya fever Arthralgia" or "Chikungunya fever arthrodynia" or "Chikungunya fever Arthralgia" or "CHIKV transmiss*" or "CHIKV vector" or "CHIKV host" or "CHIKV circulation") ) ) and (TITLE-ABS-KEY ( ("Transmiss*" or "Spread*" or "Circulat*" or "Distribut*" or "Disseminat*" or "Propagat*" or "Contagion*" or "Communicat*" or "Diffus*" or "Dispers*" or "Expans*" or "Outbreak*" or "Epidemic*" or "Pandemic*" or "Prevalen*" or "Inciden*" or "Occurren*" or "Emergen*" or "Clon*" or "Persisten*" or "Infect*") ) )

**Web of Science**

TS=(Climat* or Climate or Changing climate or Global warming or Climate variability or Climate fluctuation or Rising temperature or Temperature or Precipitation or Extreme weather or Extreme event or Extreme temperature or Heat wave or Drought or Flood or Sea level rise or CO2 concentration or Greenhouse effect or Greenhouse gas or Anthropogenic forcing or Natural forcing) and TS=(Chikungunya Virus or Chikungunya Fever or CHIKV or Chikungunya disease or Chikungunya virus infection or CHIKV infection or Arthralgia, Chikungunya fever or Chikungunya fever Arthralgia or Chikungunya fever arthrodynia or Chikungunya fever Arthralgia or CHIKV transmiss* or CHIKV vector or CHIKV host or CHIKV circulation) and TS=(Transmiss* or Spread* or Circulat* or Distribut* or Disseminat* or Propagat* or Contagion* or Communicat* or Diffus* or Dispers* or Expans* or Outbreak* or Epidemic* or Pandemic* or Prevalen* or Inciden* or Occurren* or Emergen* or Clon* or Persisten* or Infect*)

**EBSCOhost**

SU(Climat* or Climate or Changing climate or Global warming or Climate variability or Climate fluctuation or Rising temperature or Temperature or Precipitation or Extreme weather or Extreme event or Extreme temperature or Heat wave or Drought or Flood or Sea level rise or CO2 concentration or Greenhouse effect or Greenhouse gas or Anthropogenic forcing or Natural forcing) and SU(Chikungunya Virus or Chikungunya Fever or CHIKV or Chikungunya disease or Chikungunya virus infection or CHIKV infection or Arthralgia, Chikungunya fever or Chikungunya fever Arthralgia or Chikungunya fever arthrodynia or Chikungunya fever Arthralgia or CHIKV transmiss* or CHIKV vector or CHIKV host or CHIKV circulation) and SU(Transmiss* or Spread* or Circulat* or Distribut* or Disseminat* or Propagat* or Contagion* or Communicat* or Diffus* or Dispers* or Expans* or Outbreak* or Epidemic* or Pandemic* or Prevalen* or Inciden* or Occurren* or Emergen* or Clon* or Persisten* or Infect*)

**4. Search results**

| Database | Number of documents found |
| --- | --- |
| Pubmed | 308 |
| Web of science | 1125 |
| Scopus | 757 |
| EBSCOhost | 146 |
| Total | 2336 |

Records after duplicates removed: 1454
