# Supplementary material for: Impact of climate change on the global circulation of chikungunya virus: current evidence, future projections, and adaptation strategies
Source: Infect Dis Poverty. 2026 Jul 24;15:83. doi: 10.1186/s40249-026-01480-3 (PMC13397683; doi:10.1186/s40249-026-01480-3)
Supplement: Supplementary file 5 — Supplementary Material 5 [file 40249_2026_1480_MOESM5_ESM.docx]

**Additional file 5. The optimal transmission temperature reported in each study**

**Table 1 Optimal transmission temperatures proposed by various authors**

| **Study authors** | **Optimal transmission temperature** |
| --- | --- |
| Velu, RM et al.^1^ | 25 °C-27 °C |
| Doeurk, Bros et al.^2^ | 25 °C-30 °C |
| Caldwell JM et al.^3^ | 20 °C-30 °C |
| Tewari, P et al.^4^ | 17.64°C-34.50°C |
| Kakarla, SG et al.^5^ | 20 °C-34 °C |
| Steindorf, V et al.^6^ | 22°C-27 °C |
| Huber, JH et al.^7^ | 25°C-35°C |
| Carbajo, A et al.^8^ | 26°C-28°C |
| Freitas, LP et al.^9^ | 26°C-29°C |
| Dias, R et al.^10^ | 25°C-35°C |

**References**

1. Velu RM, Kwenda G, Bosomprah S, et al. Ecological Niche Modeling of Aedes and Culex Mosquitoes: A Risk Map for Chikungunya and West Nile Viruses in Zambia. Viruses. 2023;15(9):1900. doi:10.3390/v15091900

2. Doeurk B, Leng S, Long Z, Maquart PO, Boyer S. Impact of temperature on survival, development and longevity of *Aedes aegypti* and *Aedes albopictus* (Diptera: Culicidae) in Phnom Penh, Cambodia. Parasit Vectors. 2025;18(1):362. doi:10.1186/s13071-025-06892-y

3. Caldwell JM, LaBeaud AD, Lambin EF, et al. Climate predicts geographic and temporal variation in mosquito-borne disease dynamics on two continents. Nat Commun. 2021;12(1):1233. doi:10.1038/s41467-021-21496-7

4. Tewari P, Ma P, Gan G, et al. Non-linear associations between meteorological factors, ambient air pollutants and major mosquito-borne diseases in Thailand. PLoS Negl Trop Dis. 2023;17(12):e0011763. doi:10.1371/journal.pntd.0011763

5. Kakarla SG, Mopuri R, Mutheneni SR, et al. Temperature dependent transmission potential model for chikungunya in India. Sci Total Environ. 2019;647:66-74. doi:10.1016/j.scitotenv.2018.07.461.

6. Steindorf V, KB HM, Stollenwerk N, et al. Forecasting invasive mosquito abundance in the Basque Country, Spain using machine learning techniques. Parasit Vectors. 2025;18(1):109. doi:10.1186/s13071-025-06733-y

7. Huber JH, Childs ML, Caldwell JM, Mordecai EA. Seasonal temperature variation influences climate suitability for dengue, chikungunya, and Zika transmission. PLoS Negl Trop Dis. 2018;12(5):e0006451. doi:10.1371/journal.pntd.0006451

8. Carbajo AE, Vezzani D. Waiting for chikungunya fever in Argentina: spatio-temporal risk maps. Mem Inst Oswaldo Cruz. 2015;110(2):259-262. doi:10.1590/0074-02760150005

9. Freitas LP, Schmidt AM, Cossich W, Cruz OG, Carvalho MS. Spatio-temporal modelling of the first chikungunya epidemic in an intra-urban setting: the role of socioeconomic status, environment and temperature. PLoS Negl Trop Dis. 2021;15(6):e0009537. doi:10.1371/journal.pntd.0009537

10. Dias R, Leite MPC, Corrêa-do-Nascimento GS, et al. Impact of Thermal Variation on Egg Hatching and the Life Cycle of Aedes (Protomacleaya) terrens (Diptera: Culicidae) in a Laboratory Environment. Life (Basel). 2025;15(7):1038. doi:10.3390/life15071038
