# Supplementary material for: Impact of climate change on the global circulation of chikungunya virus: current evidence, future projections, and adaptation strategies
Source: Infect Dis Poverty. 2026 Jul 24;15:83. doi: 10.1186/s40249-026-01480-3 (PMC13397683; doi:10.1186/s40249-026-01480-3)
Supplement: Supplementary file 6 — Supplementary Material 6 [file 40249_2026_1480_MOESM6_ESM.docx]

**Additional file 6. Classification of adaptation strategies against CHIKV risks in response to climate change**

**Table 1 Summarizing the 12 sub-sectors of adaptation strategies**

| **Main Domain** | **Sub-sector** | **Corresponding Tier in the Framework** |
| --- | --- | --- |
| **Monitor risk and anticipate** | Integrated vector-virus surveillance | Mid-tier (Pre-emptive predicting & alerting) |
|  | Climate-based early warning systems | Mid-tier (Pre-emptive predicting & alerting) |
| **Strengthen vector control** | Resistance management | Top-tier (Reactive crisis response) |
|  | Integrated vector management | Top-tier (Reactive crisis response) |
| **Reduce epidemic risk** | Urban planning & water management | Bottom-tier (Foundational adaption & long-term surveillance) |
|  | Community behavior change | Bottom-tier (Foundational adaption & long-term surveillance) |
| **Enhance innovation and new approaches** | Novel biocontrol technologies & digital health | Bottom-tier (Foundational adaption & long-term surveillance) |
|  | Vaccine deployment | Bottom-tier (Foundational adaption & long-term surveillance) |
| **Prevent and prepare for pandemics** | Differential diagnosis capacity | Top-tier (Reactive crisis response) |
|  | Health system surge capacity | Top-tier (Reactive crisis response) |
| **Build a coalition of partners** | Cross-border governance | Bottom-tier (Foundational adaption & long-term surveillance) |
|  | One health collaboration | Bottom-tier (Foundational adaption & long-term surveillance) |
